# Retraction: Inhibition of mitochondrial fission as a novel therapeutic strategy to reduce mortality upon myocardial infarction

**DOI:** 10.1042/CS20180671_RET

**Published:** 2025-08-13

This commentary is being retracted from *Clinical Science* at the request of the authors and the Editorial Board. This follows the retraction of the article “Balancing mitochondrial dynamics via increasing mitochondrial fusion attenuates infarct size and left ventricular dysfunction in rats with cardiac ischemia/reperfusion injury” Maneechote et al., Clin Sci (Lond) (2019) 133 (3): 497–513 (DOI:10.1042/cs20190014), upon which this commentary was based. It was noted that multiple protein bands in certain figures appeared similar with rearrangement, and several western blots contained signs of digital splicing.

The authors of this commentary had no involvement in the retracted research article by Maneechote et al., and the retraction of this associated commentary is based solely on the Journal having lost confidence in the validity of the scientific data presented in the original research paper. The authors were contacted and agree with the Journal’s decision.

